# National audit of the structure and function of Australian residential care medication advisory committees

**DOI:** 10.1111/ajag.70048

**Published:** 2025-05-22

**Authors:** Amanda J. Cross, Brooke Blakeley, Helen V. Dowling, Kate Laver, Terry P. Haines, Sarah N. Hilmer, Atish Manek, Alexandra Bennett, Angelita Martini, Lyntara Quirke, Mary Ann Kulh, J. Simon Bell

**Affiliations:** ^1^ Centre for Medicine Use and Safety, Faculty of Pharmacy and Pharmaceutical Science Monash University Melbourne Victoria Australia; ^2^ Australian Commission on Safety and Quality in Health Care Sydney New South Wales Australia; ^3^ College of Nursing and Health Sciences Flinders University Adelaide South Australia Australia; ^4^ School of Primary and Allied Health Care, Faculty of Medicine Nursing and Health Sciences Monash University Melbourne Victoria Australia; ^5^ Kolling Institute of Medical Research, Northern Sydney Local Health District and Faculty of Medicine and Health The University of Sydney Sydney New South Wales Australia; ^6^ Department of General Practice, Faculty of Medicine Nursing and Health Sciences Monash University Melbourne Victoria Australia; ^7^ New South Wales Therapeutic Advisory Group Sydney New South Wales Australia; ^8^ Faculty of Medicine and Health, Sydney Pharmacy School University of Sydney Sydney New South Wales Australia; ^9^ Calvary Health Care Sydney New South Wales Australia; ^10^ University of Western Australia Perth Western Australia Australia; ^11^ Consumer Representative Dementia Australia Canberra Australian Capital Territory Australia; ^12^ School of Medicine and Psychology Australian National University Acton Australia

**Keywords:** clinical audit, clinical governance, long‐term care, medication therapy management, quality improvement

## Abstract

**Objective:**

All Australian residential care facilities are recommended to have access to a medication advisory committee (MAC) to provide governance of medication management. The objective was to explore the structure and function of Australian MACs.

**Methods:**

A national 43‐item survey of MACs was conducted from November 2023 to January 2024. The survey was adapted from the Australian Government Department of Health and Aged Care Audit Tool and Checklist for a Medication Advisory Committee (Audit Tool). All MAC representatives were recruited using a comprehensive and purposive strategy including the Department of Health and Aged Care newsletter, professional organisations, social media and professional contacts. Outcomes included self‐reported MAC structure and function across four key roles as per the Audit Tool, including policy development, risk management, education and quality improvement.

**Results:**

Responses were received from 120 MACs covering 642 residential care facilities (24% of Australian residential care facilities) in all Australian states and mainland territories. The MACs provided oversight to a median (IQR) 116 (61–196) beds/residents and a median (IQR) 1 (1–4) facilities. Over half (58%) of MACs were multidisciplinary (nursing, pharmacist and prescriber representation). More than half of MACs reported performing all functions listed in the Audit Tool relating to policy development (59%) and risk management (53%). Only 41% and 28% of MACs reported they performed all functions in the Audit Tool related to education and quality improvement, respectively.

**Conclusion:**

There is extensive heterogeneity in the structure and function of MACs with scope for MACs to become more multidisciplinary, identify staff training needs and proactively lead quality improvement.

**Practice impact**

Medication Advisory Committee (MAC) structure and function varies widely. Over half were multidisciplinary (58%), and 28% reported performing all functions listed in the audit tool for quality improvement. Future initiatives should focus on ensuring MACs are multidisciplinary and are implementing and evaluating quality improvement strategies, particularly in non‐metropolitan areas.

## 1 INTRODUCTION

The Australian Government's Royal Commission into Aged Care Quality and Safety highlighted opportunities for better medication management in residential care facilities.^1^ Medication Advisory Committees (MACs) are a key strategy to promote medication safety in residential care facilities.^2^, ^3^ A MAC is a ‘multidisciplinary committee that provides overarching governance of medication management to ensure the judicious, appropriate, safe and quality use of medications’.^4^ ^p. 3^ All Australian residential care facilities are recommended to have access to a MAC. MACs operate at a system‐level to support evidence‐based practice. Australian MACs are similar to international quality circles (multidisciplinary peer review groups) that support primary health‐care professionals,^5^, ^6^ and hospital‐based drug and therapeutics committees.^7^

MACs have existed for over 25 years, but there has been limited research into their structure and function.^8^ In 2017, MACs were identified as a key intervention to manage increasing polypharmacy.^2^ In 2020, a qualitative study explored the structure and function of four MACs that supported 27 residential care facilities in the state of Victoria.^9^ This study, conducted in partnership with the Victorian Government Department of Health and Human Services, made 12 recommendations to optimise MAC structure and function and identified opportunities for MACs to transition from a reactive to a proactive model of quality improvement. However, there have been no studies in other states or with a larger sample size.

In 2022, the then Australian Government Department of Health and Aged Care (now known as Australian Government Department of Health, Disability and Ageing) published updated ‘Guiding Principles for Medication Management in Residential Aged Care Facilities’ (the Guiding Principles).^10^ This included a ‘User Guide: Role of a Medication Advisory Committee’^4^ and ‘Audit Tool and Checklist for a Medication Advisory Committee’ (Audit Tool).^11^ The Audit Tool was designed to support self‐reflection by residential care provider organisations to identify improvement opportunities. However, there were no national baseline data on the structure and function of MACs for residential care provider organisations to benchmark against. Benchmarking self‐audit results against national data may help to drive quality improvement. The objective of this study was to explore the current structure and function of Australian MACs.

## 2 METHODS

A national cross‐sectional online survey was conducted in November 2023 to January 2024. This manuscript has been reported as per the Checklist for Reporting of Survey Studies (CROSS).^12^

### 2.1 Survey development

The survey instrument was based on the ‘User Guide: Role of a Medication Advisory Committee’^4^ and ‘Audit tool and checklist for a Medication Advisory Committee’.^11^ The survey included all questions in the Audit Tool, but several multi‐part questions in the Audit Tool were divided into separate survey items and additional response options were added for some items (i.e. in addition to yes/no). The content validity and face validity of the survey instrument were evaluated by a multidisciplinary panel comprising representatives from two residential care provider organisations. Minor amendments were made to the survey based on the panel feedback, such as improving clarity of some questions by providing definitions of key concepts.

The survey instrument was divided into two sections and included closed‐ended (e.g. dichotomous, multiple choice and multi‐answer questions) and open‐ended free‐text items. Section A related to establishing and implementing MACs and included six items from the Audit Tool and 10 items on MAC and residential care facility demographics and structure. Section B related to the four roles of MACs outlined in the Audit Tool: (1) develop and endorse policies, procedures and guidelines and advise on legislation and standards (three items), (2) advise on risk management systems associated with medication management (seven items), (3) identify education and training needs for medication management (eight items) and (4) monitor effectiveness and performance as well as implement quality improvement strategies for medication management (nine items).

### 2.2 Participants and sample size

The survey instrument was designed for completion by MAC chairs or members employed (e.g. registered nurses) or contracted (e.g. general practitioners, accredited pharmacists [now known as credentialled pharmacists], and community pharmacists) by residential care provider organisations. In the absence of a register or list of Australian MACs, we adopted a broad but purposive sampling strategy to elicit responses from MAC chairs or members in metropolitan and non‐metropolitan areas across all Australian states and mainland territories. The survey was open for a 9‐week period. The study did not involve hypothesis testing and so we did not perform an a priori sample size calculation. However, we aimed to recruit participants that represented MACs covering more than 10% of Australia's 2639 residential care facilities.^13^

### 2.3 Recruitment

Prospective participants were identified via expression of interest through professional (Aged and Community Care Providers Association, Pharmaceutical Society of Australia, Society of Hospital Pharmacists Australia [now known as Advanced Pharmacy Australia]), research (Residential Aged Care Research Network: RACReN) and government channels (‘Your Aged Care Update’ Department of Health and Aged Care newsletter), social media (Facebook, LinkedIn, X/Twitter) or via direct contact to professional contacts of the project investigators.

Prospective participants were asked to complete an online expression of interest (EOI) form. Those who completed the EOI form were sent the explanatory statement. A member of the research team reviewed each EOI to ensure that another representative of the same MAC had not already participated. Written informed consent was then obtained from all participants via email. Participants were provided with the link to complete the online survey and a unique study identification number to input into the online survey. The survey was administered using the Research Electronic Data Capture (REDCap) electronic data capture tool. Participants who were members of multiple MACs were able to complete the survey for each MAC, provided a different member of that MAC had not already completed the survey.

### 2.4 Data collection and analysis

Data were collected and managed using the REDCap electronic data capture tool hosted and managed by Helix (Monash University). REDCap is a secure, web‐based software platform designed to support data capture for research studies.^14^, ^15^ Data were imported to Microsoft® Excel (Microsoft Corporation, 2024, version 16.81). Duplicate survey entries (same survey ID) were removed.

Descriptive statistics (frequencies and percentages for categorical and ordinal data, median and interquartile range (IQR) for discrete data) were used to describe demographics, structure and function of MACs. Exploratory bivariate analyses were performed using Pearson's *χ* ^2^ test to compare demographics of MACs (metropolitan vs. non‐metropolitan, multidisciplinary vs. non‐multidisciplinary and 1:1 MAC:residential care facility ratio vs. 1:>1 MAC:residential care facility ratio). Continuity correction was reported for 2 × 2 analyses. Data analysis was conducted using the Statistical Package for Social Sciences (SPSS, version 29.0; IBM Corp. Armonk, USA).

### 2.5 Ethics approval

Ethics approval was granted by the Monash University Human Research Ethics Committee (ID 39206).

## 3 RESULTS

### 3.1 Survey responses

A total of 113 EOIs were received from MAC chairs and members and 71 (63%) consented to participate. Complete survey responses were received from 71 MAC chairs or members (100% of consenting participants). These respondents represented 120 MACs that supported 642 unique residential care facilities (24% of all Australian residential care facilities).

### 3.2 Medication Advisory Committee demographics and structure

The 120 MACs operated across all Australian states and mainland territories, with geographic distribution similar to that of national Australian residential care services (Table 1). More than half were from non‐metropolitan regions (78, 65%) and from either independent not‐for‐profit (59, 49%) or private residential care provider organisations (45, 38%). A proportion of MACs also oversaw community services (16, 13%), hospital (including inpatient, acute or sub‐acute) services (6, 5%) and disability support services (5, 4%).

**TABLE 1:** Demographics of participating MACs vs. demographics of Australian residential care services.

Demographics	Participating MACs, *n* (%)	Australian residential care services, *n* (%)^a^
State and Territories		
Victoria	57 (48)	748 (28)
New South Wales	40 (33)^b^	835 (32)
South Australia	14 (12)^b^	229 (9)
Queensland	11 (9)^b^	468 (18)
Western Australia	9 (8)^b^	249 (9)
Australian Capital Territory	5 (4)^b^	27 (1)
Tasmania	3 (2)^b^	71 (3)
Northern Territory	2 (2)^b^	12 (1)
Geographic region		
Metropolitan	74 (62)^b^	1653 (63)^c^
Regional	51 (43)^b^	439 (17)^c^
Rural	20 (17)^b^	507 (19)^c^
Remote	7 (6)^b^	40 (2)^c^
Organisation funding type		
Independent not‐for‐profit	59 (49)	123,382 (56)^d^
Private	45 (38)	90,159 (41)^d^
Public	13 (11)	7926 (4)^d^
Other	3 (3)	–

^a^ Australian demographics defined as residential care service provision as at 30th June 2023 as reported on GEN‐agedcaredata.gov.au.

^b^ Not mutually exclusive.

^c^ Mutually exclusive, metropolitan defined as Monash Model (MM) 1, regional MM2–3, rural MM4–5, remote MM6–7.

^d^ Reported as number of resident places not number of residential care facilities.

**FIGURE 1:**
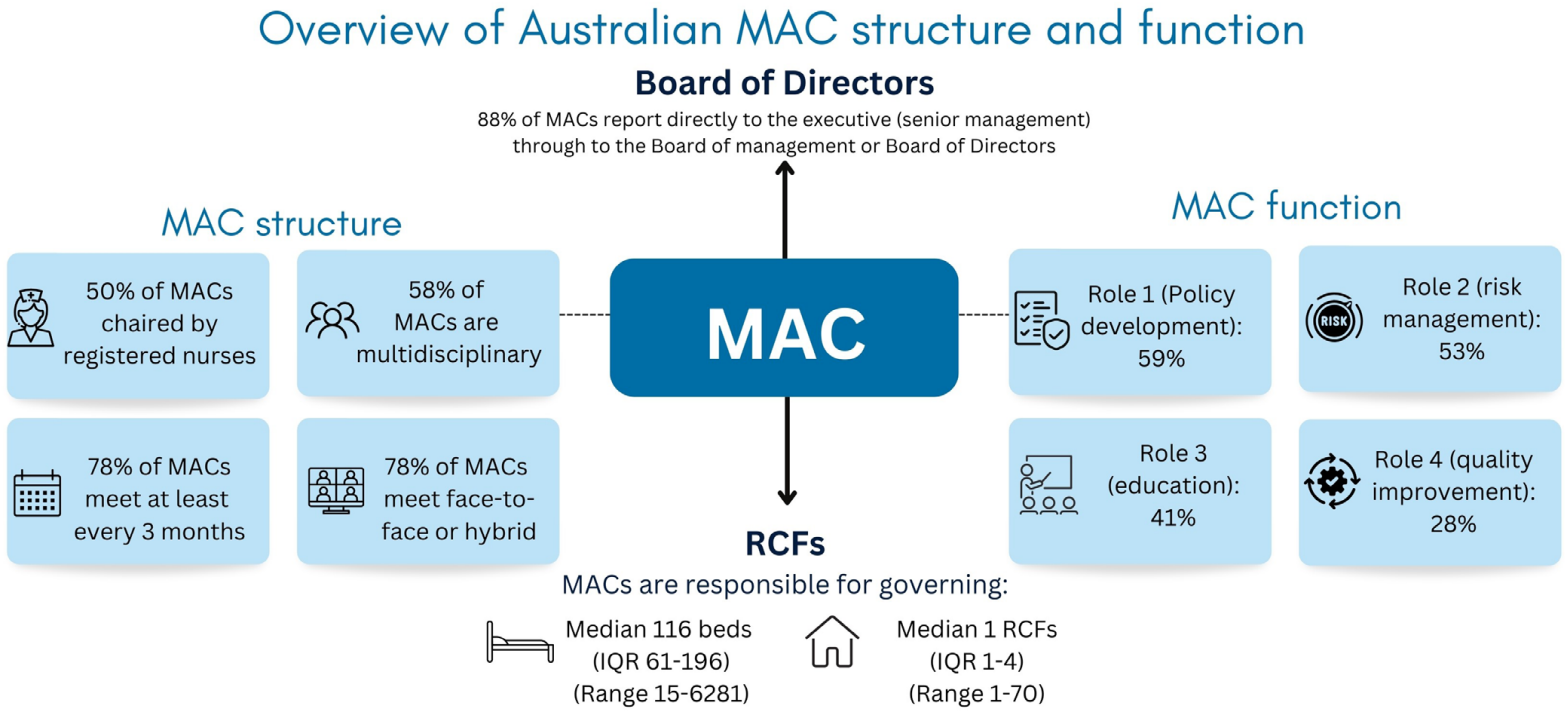
Overview of Australian medication advisory committee (MAC) structure and function. Summary of results from national audit of 120 Australian MACs. MAC function results refer to percentage of MACs that reported doing all functions under the four roles as outlined in the Australian Government Department of Health and Aged Care Audit Tool and Checklist for a Medication Advisory Committee. IQR, interquartile range; MAC, medication advisory committee; RCFs, residential care facilities.

MACs oversaw from 15 beds to 6281 beds and from 1 to 70 residential care facilities (Figure 1). The majority (105, 88%) of MACs reported directly to executive (senior management) through to the board or management (Table 2).

**TABLE 2:** Structure of Australian MACs.

Structure characteristics	*n* (%), unless stated
Number of RCFs the MAC oversees, median (IQR)	1 (1–4)
Number of Beds MAC oversees, median (IQR)	116 (61–196)
Number of Beds MAC oversees	
1–50	19 (16)
51–100	33 (28)
101–150	23 (19)
151–200	11 (9)
200+	28 (23)
Didn't answer	6 (5)
Number of members of MAC, median (IQR)	7 (5, 11)
Number of people at last MAC, median (IQR)	6 (4, 9)
Members of the MAC	
Accredited medication review and/or QUM pharmacist	111 (93)
Registered nurse	105 (88)
Senior management	103 (86)
Community pharmacist	100 (83)
General practitioner	70 (58)
Administrator or secretariat	35 (29)
Nurse practitioner	19 (16)
Onsite/embedded pharmacist	11 (9)
Resident or resident advocate	10 (8)
Geriatrician	6 (5)
Other^a^	23 (19)
Chair of the MAC	
Registered nurse	60 (50)
Pharmacist	20 (17)
Other^b^	40 (33)
Reports to Executive/Board, yes	105 (88)
Has an accountability framework	95 (79)
Has an agreed and approved terms of reference (ToR) document	96 (80)
Has a ToR that has been reviewed in the last 12 months^c^	82 (68)
Frequency of MAC meetings	
More often than every 3 months	5 (4)
Every 3 months	89 (74)
Every 6 months	24 (20)
Ad hoc	2 (2)
Format of meetings	
Face‐to‐face	52 (43)
Virtual	27 (23)
Hybrid	41 (34)

Abbreviations: IQR, Interquartile range; MAC, Medication advisory committee; QUM, Quality use of medication; RCF, Residential care facility; ToR, Terms of reference.

^a^ *n* = 23 MACs indicated that one or more ‘other’ members attended their MAC.

^b^ Other members described in full text included, but were not limited to, care managers, facility managers and clinical care coordinators.

^c^ Indicates a question that only appeared if the preceding question was answered as ‘yes’, but denominators have been presented as *n* = 120.

Most MACs met every 3 months (89, 74%) in face‐to‐face (52, 43%) or hybrid face‐to‐face/virtual (41, 34%) formats. The median (IQR) number of MAC members was 7 (5–11), and the reported attendance at the most recent MAC meeting ranged from 43% to 100%. Over half (70, 58%) of the MACs were multidisciplinary (i.e. had nurse, pharmacist and prescriber members). The most common health professional members were accredited medication review and/or Quality Use of Medication (QUM) pharmacists (111, 93%), registered nurses (105, 88%), community pharmacists (100, 83%) and general practitioners (70, 58%). Half the MACs were chaired by registered nurses.

### 3.3 Role 1: Develop and endorse policies and procedures, and advise on legislation and standards

Over half (71, 59%) of MACs reported performing all recommended functions within the policy development role listed in the Audit Tool. More than three‐quarters of MACs reported performing functions relating to developing and endorsing policies, procedures and guidelines relating to medication management (94, 78%), including ensuring that the policies, procedures and guidelines were accessible to staff (110, 92%) and that there was a communication strategy for new, revised or updated documents (91, 76%) (Table 3). When the MAC did not perform these functions, other clinical governance teams or management/head office were reported to be responsible for the functions. No significant associations were identified between MACs who reported they performed policy development functions and the geography of the MAC, multidisciplinary nature of the MAC or the MAC: residential care facilities ratio (Table S1).

**TABLE 3:** Scope and functions of Australian MACs, as per roles described in the MAC User Guide and Audit Tool.^4^, ^11^

Function	Yes, *n* (%)
**Role 1: develop and endorse policies, procedures and guidelines and advise on legislations and standards**	**71 (59)** ^b^
Develop/endorse policies/procedures/guidelines representing all elements of medication management	94 (78)
Ensure policies/procedures/guidelines are accessible to all RCF healthcare professionals and external healthcare professionals	110 (92)
Documented communication strategy for new, revised or updated policies/procedures/guidelines	91 (76)
**Role 2: advise on risk‐management systems and the management of risks associated with medication management**	**64 (53)** ^c^
Informing and updating risk assessments and risk management system associated with medication management	100 (83)
Collaboratively develop strategies to control, reduce or eliminate medicines‐related risks	117 (98)
Regularly review need for RCF healthcare professional education and training on medication management and risk mitigation strategies	106 (88)
Reviewed updated Guiding Principles for Medication Management in Residential Aged Care Facilities	76 (63)
Ensures adherence with Guiding Principles for Medication Management in Residential Aged Care Facilities^a^	75 (63)
**Role 3: identify education and training needs for medication management**	**49 (41)** ^d^
Support provision and access to education and training on medication management	112 (93)
The education and training provided is based on the specific needs of the RCF healthcare professionals, the facility and those receiving care^a^	109 (91)
Support and provide input into an internal and/or external learning and development program^a^	94 (78)
Implement processes to assess competency and training needs of RCF workforce regarding medication management	70 (58)
Implement process to perform risk assessment to inform training needs and priorities for the RCF workforce regarding medication management	83 (69)
Implement process to develop or provide access to training and education resources to meet the needs of the RCF workforce regarding medication management	97 (81)
Implement process to use ongoing education programs to supplement existing knowledge and skills of the multidisciplinary workforce	93 (78)
**Role 4: monitor effectiveness and performance as well as the implementation of quality improvement strategies for medication management**	**34 (28)** ^e^
Develop policies, procedures and guidelines for systematic evaluation of QUM	82 (68)
Evaluate existing QUM strategies	102 (85)
Proactive and responsive to medication management issues and risks	115 (96)
Develop action plan in response to medication management issues and risks^a^	90 (75)
Utilise a pharmacist to support QUM activities	116 (97)
Review medicine utilisation trends and usage patterns	100 (83)
Measure and improve individuals' experience with medication management	57 (48)
Plan and drive QUM and medication safety initiatives	110 (92)

Abbreviations: MAC, Medication advisory committee; QUM, Quality use of medication; RCF, Residential care facility.

^a^ Question only appeared if the preceding question was answered as ‘yes’, but denominators presented as *n* = 120.

^b^ Represents MACs that completed all three functions under the policy development role as described in the Audit Tool.

^c^ Represents MACs that completed all five functions under the risk management role as described in the Audit Tool.

^d^ Represents MACs that completed all seven functions under the education role as described in the Audit Tool.

^e^ Represents MACs that completed all eight functions under the quality improvement role as described in the Audit Tool.

### 3.4 Role 2: Advise on risk‐management systems and the management of risks associated with medication management

Over half (64, 53%) of MACs reported performing all recommended functions within the risk management role in the Audit Tool. Advising on risk management, through collaborative development of strategies to control, reduce or eliminate medicine‐related risks was the most common reported function of MACs (117, 98%) (Table 3). Risk management activities were informed by internal audits (95, 79%), National Quality Indicator Program data (90, 75%), electronic medication incident reporting systems (84, 70%), medication use patterns and trends (84, 70%), quality improvement strategies (83, 69%) and serious incident response scheme (SIRS) reports (83, 69%). Twenty MACs (18%) reported they were not responsible for informing and updating risk assessments and the risk management system associated with medication management. More than half of MACs had reviewed and self‐reported being adherent with the ‘Guiding Principles for Medication Management in Residential Aged Care Facilities’ document (75, 63%). A further 23% (*n* = 27) reported they planned to review the Guiding Principles within the next 6 months. No significant associations were identified between MACs who reported they performed risk assessment functions and the geography of the MAC, multidisciplinary nature of the MAC or the MAC: residential care facilities ratio (Table S1).

### 3.5 Role 3: Identify education and training needs for medication management

Less than half (49, 41%) of MACs reported performing all education roles outlined in the Audit Tool. While the majority of MACs supported the provision and access to education and training on medication management (112, 93%), only half reported that the MAC had a role in implementing processes to assess workforce competency and training needs (70, 58%) (Table 3). When this was not the responsibility of the MAC, participants qualitatively described this role was commonly reported to be the responsibility of the residential care facility or the facility manager, head office or other quality, governance or education committees. Education was mostly delivered face‐to‐face (106, 88%) and via e‐learning (86, 72%). No significant associations were identified between MACs who reported they performed education functions and the geography of the MAC, multidisciplinary nature of the MAC or the MAC:residential care facilities ratio (Table S1).

### 3.6 Role 4: Monitor effectiveness and performance as well as the implementation of quality improvement strategies for medication management

One in four MACs (34, 28%) reported performing all functions within the quality improvement role as described in the Audit Tool. Almost all MACs reported being proactive and responsive to medication management issues and risks (115, 96%), and three‐quarters of those MACs (90/115, 78%) reported that they developed action plans in response to identified issues and risks. Nearly all MACs reported that they utilised a pharmacist to support QUM activities (116, 97%). The existing strategies or sources of data most used to evaluate QUM included number and type of medication incidents (98, 82%), the psychotropic register (93, 78%) and numbers of residential medication management reviews completed (90, 75%). Eighteen (15%) MACs reported they did not evaluate existing QUM strategies. Less than half of the MACs measured resident experiences with medication management (57, 48%), with most responding that this was completed by facility‐level staff (e.g. registered nurses, care staff, facility managers or pharmacists) or not at all. Being located in a metropolitan area was significantly associated with performing all quality improvement functions listed in the Audit Tool compared to non‐metropolitan MACs (32/74 vs. 2/46, *p* < .001) (Table S1).

## 4 DISCUSSION

This study represents the most comprehensive exploration of the structure and function of Australian MACs. It highlights scope for improvement in multidisciplinary membership and purpose of the MAC by ensuring current terms of reference, and opportunities for targeted interventions to ensure MACs are adherent to the Guiding Principles,^10^ are identifying education and training needs relevant to their residential care staff, and proactively implementing and evaluating quality improvement initiatives.

More than half of MACs involved a nurse, pharmacist and prescriber member. This was consistent with expert panel recommendations for MACs in residential care facilities,^9^ and with best‐practice recommendations for drugs and therapeutics committees.^7^ Encouragingly, the proportion of MACs with general practitioners (GPs) was double that reported among a sample of regional and rural MACs in 2020.^9^ However, medical prescriber representation was still the lowest of the three core disciplines. Local and system‐level strategies to promote GP engagement in residential care, including overcoming administrative, time and financial barriers, are needed.^16^ Local strategies could include ensuring the MAC operates effectively, at a time and in a format convenient to all personnel, and that the agenda is focused on governance and clinical risk management and not day‐to‐day operational, administrative or bureaucratic concerns.^9^, ^16^ At a system level, new ‘General Practice in Aged Care’ incentives commenced in July 2024 may facilitate increased engagement by GPs in MACs.^17^ Increasing awareness of the potential for MAC‐led quality improvement, through sharing of successes, adapting knowledge from hospital based drugs and therapeutics committees and increased research on the role of MACs may also drive necessary organisational and cultural changes.^9^

Two‐thirds of MACs reported having up‐to‐date terms of reference, and three‐quarters were chaired by an internal member of staff. This suggests there may be scope to optimise MAC operation by improving clarity regarding the role of the MAC in areas such as policy development, risk management, education and quality improvement. As identified in the Royal Commission into Aged Care Quality and Safety, ‘better system governance is crucial to the reform of aged care’.^1^ ^p. 82^ Expert panel recommendations for MACs support appointing an independent chair of the MAC, such as a pharmacist, for the purpose of ensuring good clinical governance, accountability and transparency.^9^

Most MACs reported they were proactive and responsive to medication management issues. However, developing action plans, implementing and monitoring the effectiveness of quality improvement initiatives were among the least reported functions. This is consistent with previous findings that showed that while MAC members attached a high priority to preventing medicine‐related harm, the activities of MACs were often reactive and quality indicator data was just tabled at MAC meetings rather than actively discussed and used.^9^ These findings suggest there may be opportunities to optimise the function of MACs to better support continuous quality improvement that is tailored to the local needs, resources and challenges of their specific residential care facility. The need to monitor performance and address emerging issues was also highlighted in the Final Report of the Royal Commission into Aged Care Quality and Safety.^1^ Residential care provider organisations and MACs could consider utilising nurse or pharmacist change champions or knowledge brokers to support implementation and reporting back to the MAC.^18^, ^19^, ^20^ These individuals are important facilitators of success and may help to translate evidence and guidelines into practice.^19^

A key strength of this study was the diverse sample of MACs from all states and mainland territories of Australia. Responses were received from MACs representing nearly one‐quarter of all Australian residential care facilities. Responses were also received from not‐for‐profit, public and private residential care provider organisations. Our sample included MACs from both metropolitan and non‐metropolitan areas. This study aligns with key Australian Government initiatives and measured the real‐world impact of the implementation of new guidance for MACs.^4^, ^10^, ^11^ Potential limitations include the recruitment methodology. In the absence of a register or comprehensive list of Australian MACs, we employed a broad recruitment strategy. This maximised the number of MACs, but we were not able to directly calculate a response rate. It is possible that well‐functioning MACs were more likely to participate (self‐selection bias). The study relied on self‐reporting, and it is possible that MAC representatives over‐ or under‐estimated each role/function. The high proportion of MACs that involved accredited pharmacists may reflect recruitment of MAC representatives through pharmacist organisations and Facebook groups. This may have influenced the prevalence of reporting certain functions of the MACs. For these reasons, the results of the study may not be generalisable to all MACs in Australia.

## 5 CONCLUSIONS

MACs are diverse in their structure and function, and operate largely in accordance with Australian Government recommendations. However, opportunities exist for MACs to have a greater role in planning, implementing and monitoring quality improvement initiatives. Optimising the structure of MACs, including ensuring multidisciplinary representation and up‐to‐date terms of reference, may be important first steps. Future work should explore the impact of the variation in structure and function of MACs on resident and medication management outcomes, and evaluate the effectiveness and cost‐effectiveness of interventions designed to optimise the function of MACs.

## FUNDING INFORMATION

This study was supported by the Commonwealth of Australia represented by the Department of Health and Aged Care Medical Research Future Fund (MRFF) Quality, Safety and Effectiveness of Medicine Use by Pharmacists, 2022 grant (grant ID: MRFMMIP000025). AJC is supported by an NHMRC Emerging Leadership 1 grant (APP2009633).

## CONFLICT OF INTEREST STATEMENT

AJC has received grant funding or consulting funds from the Medical Research Future Fund, Dementia Australia Research Foundation and Pharmaceutical Society of Australia. All grants and consulting funds were paid to the employing institution. AJC is also a board director on the Pharmaceutical Society of Australia national board. BB declares that they have no competing interests. HVD declares that they have represented the Australian Commission on Safety and Quality in Health Care on the Project Stakeholder Group for this project, and also led a project in 2021/2022 on the development of the then Australian Government Department of Health and Aged Care (now known as Australian Government Department of Health, Disability and Ageing) 2022 edition of the Guiding Principles for Medication Management in Residential Aged Care, User Guide: Role of a Medication Advisory Committee and Audit Tool and Checklist for a Medication Advisory Committee. KL declares no COI relevant to this publication. KL has received grant funding from the National Health and Medical Research Council, the Medical Research Future Fund, the Australian Research Council, the Hospital Research Foundation, Dementia Australia and the Flinders Foundation. All grants and consulting funds were paid to the employing institution. TPH declares he is the Chair of the Board for the Australian Council of Deans of Health Sciences (ACDHS Pty Ltd). SNH declares no COI relevant to this publication. SNH has received grant funding from the National Health and Medical Research Council, Medical Research Future Fund, NSW Health, Aged Care Quality and Safety Commission unrelated to this work. All grants were paid to the administering institution. SH chairs NSW Therapeutic Advisory Group and Sydney Health Partners Geriatric Medicine Clinical Academic Group. These roles are not remunerated. AtM declares that they have no competing interests. AB has received grant funding from the Medical Research Future Fund. All funds were paid to the employing organisation. AnM declares that they have no competing interests. LQ declares that they have no competing interests. MAK declares that they have no competing interests. JSB has received grant funding or consulting funds from the National Health and Medical Research Council, Medical Research Future Fund, Victorian Government Department of Health and Human Services, Dementia Australia Research Foundation, Yulgilbar Foundation, Aged Care Quality and Safety Commission, Dementia Centre for Research Collaboration, Pharmaceutical Society of Australia, Society of Hospital Pharmacists of Australia, GlaxoSmithKline Supported Studies Program, Amgen, and several aged care provider organisations unrelated to this work. All grants and consulting funds were paid to the employing institution.

## Supporting information


Table S1

## Data Availability

The de‐identified data analysed are not publicly available, but requests to the corresponding author for the data will be considered on a case‐by‐case basis.
