# Supplementary material for: National audit of the structure and function of Australian residential care medication advisory committees
Source: Australas J Ageing. 2025 May 22;44(2):e70048. doi: 10.1111/ajag.70048 (PMC12099024; doi:10.1111/ajag.70048)
Supplement: Supplementary file 1 — Table S1 [file AJAG-44-0-s001.docx]

**Supplementary Table 1:** Exploratory bivariate analysis of function vs geography, multidisciplinary membership and MAC:RCF ratio

| **Function** | **Geography** | | | **Multidisciplinary (MD)** | | | **No. of RCFs covered by MAC** | | |
| --- | --- | --- | --- | --- | --- | --- | --- | --- | --- |
|  | **Metro, n (%) (n=74)** | **Non-Metro, n (%)**  **(n=46)** | ***p*-value** | **MD (n=70), n (%)** | **Non-MD, n (%) (n=50)** | ***p*-value** | **1 RCF, n (%) (n=75)** | **>1 RCF, n (%) (n=45)** | ***p*-value** |
| **Role 1: develop and endorse policies, procedures and guidelines and advise on legislations and standards** | **47 (64)** | **24 (52)** | **0.30** | **42 (60)** | **29 (58)** | **0.98** | **44 (59)** | **27 (60)** | **>0.99** |
| Develop/endorse policies/procedures/guidelines representing all elements of medication management | 57 (77) | 37 (80) | 0.83 | 57 (81) | 37 (74) | 0.45 | 57 (76) | 37 (82) | 0.57 |
| Ensure policies/procedures/guidelines are accessible to all RCF healthcare professionals and external healthcare professionals | 69 (93) | 41 (89) | 0.65 | 64 (91) | 46 (92) | >0.99 | 71 (95) | 39 (87) | 0.23 |
| Documented communication strategy for new, revised or updated policies/procedures/guidelines | 57 (77) | 34 (74) | 0.87 | 54 (77) | 37 (74) | 0.86 | 56 (75) | 35 (78) | 0.87 |
| **Role 2: advise on risk-management systems and the management of risks associated with medication management** | **42 (57)** | **22 (48)** | **0.44** | **34 (49)** | **30 (60)** | **0.29** | **39 (52)** | **25 (56)** | **0.85** |
| Informing and updating risk assessments and risk management system associated with medication management | 62 (84) | 38 (83) | >0.99 | 59 (84) | 41 (82) | 0.93 | 62 (83) | 38 (84) | >0.99 |
| Collaboratively develop strategies to control, reduce or eliminate medicines-related risks | 73 (99) | 44 (96) | 0.67 | 70 (100) | 47 (94) | 0.14 | 73 (97) | 44 (98) | >0.99 |
| Regularly review need for RCF healthcare professional education and training on medication management and risk mitigation strategies | 68 (92) | 38 (83) | 0.21 | 63 (90) | 43 (86) | 0.70 | 69 (92) | 37 (82) | 0.19 |
| Reviewed updated Guiding Principles for Medication Management in Residential Aged Care Facilities | 49 (66) | 27 (59) | 0.69 | 41 (59) | 35 (70) | 0.33 | 45 (60) | 31 (69) | 0.60 |
| Ensures adherence with Guiding Principles for Medication Management in Residential Aged Care Facilities^a^ | 48/49 (98) | 27/27 (100) | >0.99 | 40/41 (98) | 35/35 (100) | >0.99 | 44/45 (98) | 31/31 (100) | >0.99 |
| **Role 3: identify education and training needs for medication management** | **31 (42)** | **18 (39)** | **0.91** | **26 (37)** | **23 (46)** | **0.43** | **31 (41)** | **18 (40)** | >0.99 |
| Support provision and access to education and training on medication management | 70 (95) | 42 (91) | 0.74 | 68 (97) | 44 (88) | 0.11 | 70 (93) | 42 (93) | >0.99 |
| The education and training provided is based on the specific needs of the RCF healthcare professionals, the facility and those receiving care^a^ | 69 (93) | 40 (87) | 0.65 | 66 (94) | 43 (86) | >0.99 | 68 (91) | 41 (91) | >0.99 |
| Support and provide input into an internal and/or external learning and development program^ab^ | 60 (81) | 34 (74) | 0.49 | 55 (79) | 39 (78) | >0.99 | 59 (79) | 35 (78) | >0.99 |
| Implement processes to assess competency and training needs of RCF workforce regarding medication management | 41 (55) | 29 (63) | 0.53 | 40 (57) | 30 (60) | 0.90 | 45 (60) | 25 (56) | 0.77 |
| Implement process to perform risk assessment to inform training needs and priorities for the RCF workforce regarding medication management | 51 (69) | 32 (70) | >0.99 | 48 (69) | 35 (70) | >0.99 | 54 (72) | 29 (64) | 0.51 |
| Implement process to develop or provide access to training and education resources to meet the needs of the RCF workforce regarding medication management | 62 (84) | 35 (76) | 0.42 | 60 (86) | 37 (74) | 0.17 | 65 (87) | 32 (71) | 0.06 |
| Implement process to use ongoing education programs to supplement existing knowledge and skills of the multidisciplinary workforce | 60 (81) | 33 (72) | 0.33 | 53 (76) | 40 (80) | 0.74 | 61 (81) | 32 (71) | 0.28 |
| **Role 4: monitor effectiveness and performance as well as the implementation of quality improvement strategies for medication management** | **32 (43)** | **2 (4.3)** | **<0.001** | **23 (33)** | **11 (22)** | **0.27** | **22 (29)** | **12 (27)** | **0.92** |
| Develop policies, procedures and guidelines for systematic evaluation of QUM | 55 (74) | 27 (59) | 0.11 | 49 (70) | 33 (66) | 0.79 | 49 (65) | 33 (73) | 0.48 |
| Evaluate existing QUM strategies | 65 (88) | 37 (80) | 0.40 | 57 (81) | 45 (90) | 0.30 | 65 (87) | 37 (82) | 0.69 |
| Proactive and responsive to medication management issues and risks | 72 (97) | 43 (94) | 0.58 | 67 (96) | 48 (96) | >0.99 | 72 (96) | 43 (96) | >0.99 |
| Develop action plan in response to medication management issues and risks^a^ | 59 (80) | 31 (67) | 0.32 | 53 (76) | 37 (74) | 0.98 | 56 (75) | 34 (76) | >0.99 |
| Utilise a pharmacist to support QUM activities | 72 (97) | 44 (96) | >0.99 | 69 (83) | 47 (94) | 0.39 | 72 (96) | 44 (98) | >0.99 |
| Review medicine utilisation trends and usage patterns | 64 (87) | 36 (78) | 0.36 | 60 (86) | 40 (80) | 0.56 | 65 (87) | 35 (78) | 0.31 |
| Measure and improve individuals’ experience with medication management | 42/73 (58) | 15/46 (33) | 0.01 | 34/70 (49) | 23/49 (47) | >0.99 | 42/74 (57) | 15/45 (33) | 0.02 |
| Plan and drive QUM and medication safety initiatives | 69 (93) | 41 (89) | 0.65 | 64 (91) | 46 (92) | >0.99 | 72 (96) | 38 (84) | 0.06 |

^a^Question only appeared if the preceding question was answered as ‘yes’, but denominators presented as n=120. ^b^ Presented as dichotomous variable, both No answers reported together.
MAC, Medication advisory committee; RCF: Residential care facility; QUM, Quality use of medication
